# The diagnosis of bilateral primary renal paragangliomas in a cat

**DOI:** 10.4102/jsava.v88i0.1412

**Published:** 2017-01-24

**Authors:** Ryan B. Friedlein, Alain J. Carter, Robert D. Last, Sarah Clift

**Affiliations:** 1 Fourways Vet Hospital, Johannesburg, South Africa; 2 Faculty of Veterinary Science, Department of Companion Animal Clinical Studies, University of Pretoria, South Africa; 3 Vetdiagnostix Veterinary Pathology Services, Pietermaritzburg, South Africa; 4 Faculty of Veterinary Science, Department of Paraclinical Sciences, University of Pretoria, South Africa

## Abstract

A 9-year-old sterilised female domestic short-hair cat was referred with a history of vomiting and anorexia of 3 months’ duration. Biochemistry, full-blood counts, thoracic radiographs, feline pancreatic-specific lipase, abdominal ultrasonography and feline immunodeficiency virus/feline leukaemia virus (FIV/FeLV) SNAP tests had been performed. Mild hypochloraemia and moderate hypokalaemia were evident on initial presentation. Abdominal ultrasonography initially revealed unilateral renal nodules on the left side. These were subjected to fine-needle aspiration and cytological evaluation. A neuroendocrine tumour was suspected, and biopsies via midline coeliotomy were taken to confirm the diagnosis. Initial histopathology diagnosed primary renal carcinomas or neuroendocrine neoplasia; however, the definitive diagnosis became renal paragangliomas after immunohistochemistry and transmission electron microscopy were performed. The cat was regularly monitored with serum biochemistry parameters, blood pressure determinations, thoracic radiographs and subsequent abdominal ultrasonography. Biochemistry, radiography and blood pressures remained normal over a 24-week follow-up period, while subsequent ultrasonography revealed tumour progression in both number and size in both kidneys. Primary neuroendocrine tumours of the kidney are frequently incorrectly diagnosed as other renal tumours such as renal cell carcinoma, mesonephric tumours or undifferentiated carcinomas. This case report highlights the importance of additional testing, including immunohistochemistry and transmission electron microscopy, to obtain a definitive diagnosis of paragangliomas.

## Introduction

Paragangliomas are rare neuroendocrine tumours arising from extra-adrenal paraganglia of the autonomic nervous system (Davis et al. 1997). There are few reported cases in cats arising from various locations (Buchanan et al. 1998; Davis et al. 1997; Patnaik et al. 1990; Rizzo et al. 2008), and to the authors’ knowledge, none reported to have primary renal origin, especially of a bilateral nature. This is the first reported case of bilateral extra-adrenal primary renal paragangliomas in the cat. This case report highlights the importance of immunohistochemistry (IHC) and transmission electron microscopy (TEM) to arrive at a definitive diagnosis.

## Case presentation

A 9-year-old sterilised female domestic short-hair cat was referred with a history of intermittent vomiting and anorexia of 3 months duration. Initial investigations included a faecal floatation, urine analysis, serum biochemistry as well as a full-blood count that all yielded results within the normal range. Abdominal ultrasonography and a feline pancreatic–specific lipase test did not reveal any abnormalities. The cat was treated with prochlorperazine (Stemetil, Sanofi-Aventis) at 0.5 mg/kg per os (PO) twice per day (bid), omeprazole (Losec, AstraZeneca) at 1 mg/kg PO once per day (oid), enrofloxacin (Baytril, Bayer) at 5 mg/kg PO oid and sucralfate (Ulsanic suspension, Aspen Pharmacare) at 1 mL per 3 kg PO bid intermittently during the 3-month period. Medical therapy failed to improve the clinical condition, with vomiting and anorexia becoming more persistent. Three months after the initial investigation, a serum chemistry profile, full-blood count and urine analysis were repeated, which revealed mild hypochloraemia (98 mmol/L [normal 109 mmol/L – 122 mmol/L]) and moderate hypokalaemia (2.3 mmol/L [normal 3.5 mmol/L – 5.8 mmol/L]). In addition to the above tests, a SNAP feline leukaemia virus/feline immunodeficiency virus (FeLV/FIV) test, as well as high-definition oscillometry blood pressure readings were performed, which were with the normal range. Thoracic radiographs showed no abnormalities either. The cat was then referred to a specialist physician for further investigation.

## Management and outcome

**FIGURE 1:**
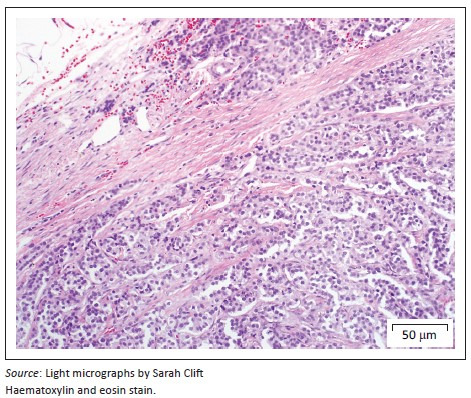
Renal paraganglioma. Partially encapsulated, multi-lobulated tumour mass consisting of packets of monotypic round to polygonal tumour cells separated by a thin fibrovascular stroma.

**FIGURE 2:**
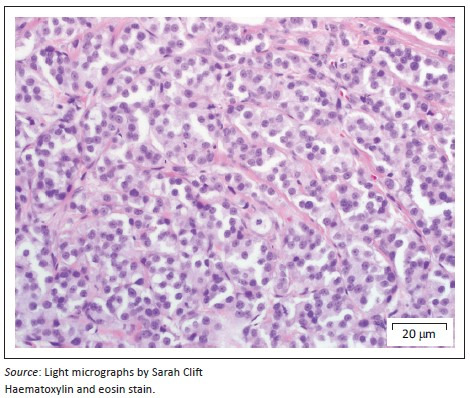
Close-up of renal paraganglioma showing neoplastic cells with round to oval nuclei containing one to three nucleoli and vacuolated, pale eosinophilic cytoplasm with indistinct cytoplasmic borders. Packets of neoplastic cells are separated by a thin fibrovascular stroma.

On presentation, the clinical parameters were normal with a fair body condition. An additional abdominal ultrasonography examination demonstrated pathology limited to the left kidney. The left kidney demonstrated two focal, round, well-demarcated 0.7 cm and 0.9-cm-diameter hypoechoic nodules in the ventral subcapsular region that bulged slightly. No nodules were seen in the right kidney. A fine-needle aspirate of one of the nodules was performed and stained with Romanowsky. Sheets and clusters of plasmacytoid round cells with eccentric and bare nuclei, cytoplasmic basophilia, moderate anisocytosis and anisokaryosis were visualised, which were suggestive of a neuroendocrine tumour. In order to obtain a more representative diagnostic sample, an exploratory laparotomy and collection of renal biopsies were conducted. Macroscopically during laparotomy, both kidneys appeared abnormal. Histopathology of both kidney biopsies revealed widespread effacement of the normal renal architecture by poorly circumscribed multi-lobulated masses that histologically appeared pseudo-glandular and were composed of nests or packets of fairly monomorphic round to polygonal neoplastic cells that were separated from adjacent packets/nests by a fine fibrovascular stroma (Figure 1). The multi-nodular tumours were partially encapsulated and variably thick, mature vascularised fibrous connective tissue septa extended from the capsule into the tumours, partially bisecting the tumours into pseudo-lobules (Figure 1). The neoplastic cells contained round to oval vesicular nuclei that were frequently centrally located and only slightly or shallowly and irregularly indented. Nuclei often contained one small centrally located nucleolus, but there were up to 2–3 peripherally located nucleoli in some cells. Nuclear chromatin varied from finely granular to coarsely stippled. Cells possessed abundant, pale, variably vacuolated, eosinophilic cytoplasm with generally indistinct cell borders (Figure 2). Nuclear pleomorphism was mild (only occasional anisokaryosis was evident in the sections), but the mitotic rate was calculated as 28 over 10 non-overlapping high-power (400×) fields, equalling 2–3 mitoses per high-power field, including atypical mitoses. Peripherally in the cell nests were rare, plump, spindle-shaped sustentacular-like cells. There was evidence of extension of neoplastic cells into the adjacent peri-renal tissues (Figure 1), but there was no convincing evidence of vascular invasion in the examined sections.

**FIGURE 3:**
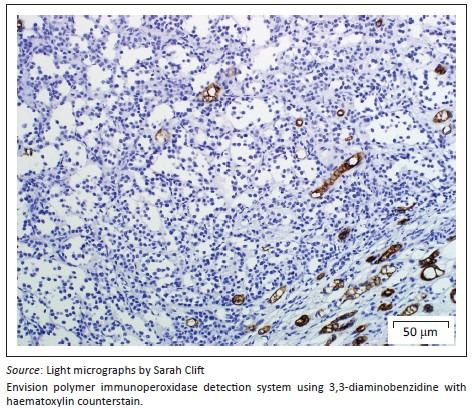
Renal paraganglioma. Neoplastic cells failed to label with the AE1/AE3 pan-cytokeratin antibody, but renal collecting tubules/ducts between the neoplastic cells labelled positive for pan-cytokeratin AE1/AE3 (which detects low- and high-molecular weight cytokeratins found in renal tubular and ductal epithelium and in urothelium).

**FIGURE 4:**
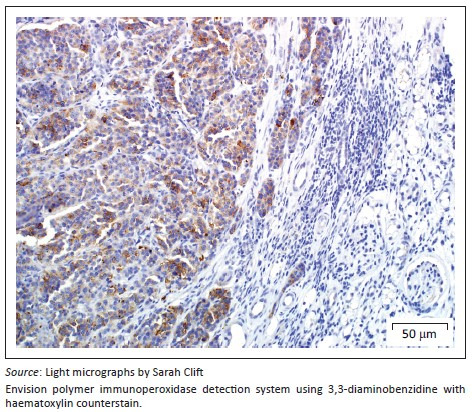
Renal paraganglioma. Neoplastic cells exhibiting strong diffuse chromogranin A-specific cytoplasmic positivity, associated with dense-core secretory vesicles/granules in neuroendocrine cells.

**FIGURE 5:**
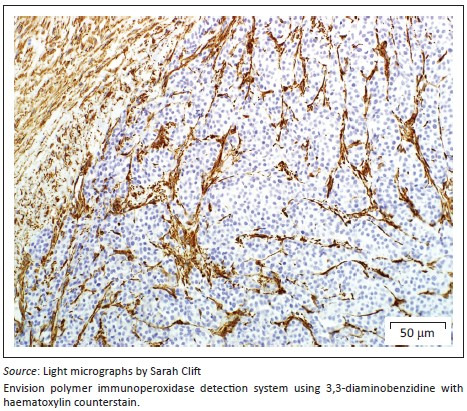
Renal paraganglioma. Fibrous connective tissue capsule and stroma labelled with vimentin (an intermediate filament protein that is expressed in mesenchymal cells), but the neoplastic cells did not, indicating that these cells were unlikely to be of mesenchymal origin.

**FIGURE 6:**
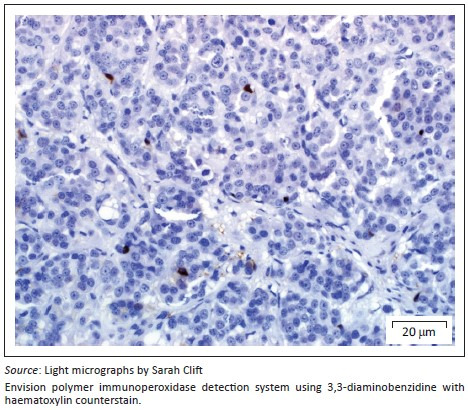
Renal paraganglioma. Rare sustentacular cells labelled in a typical nuclear and cytoplasmic pattern with the S100 antibody for calcium-binding proteins; S100 is highly specific for sustentacular cells within the normal or tumourous adrenal medulla, extra-adrenal paraganglia and autonomic ganglia.

**FIGURE 7:**
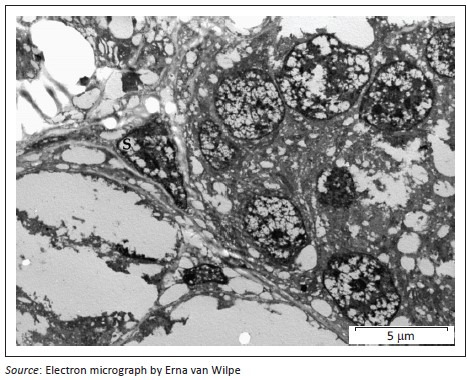
Transmission electron micrograph; renal paraganglioma. Sustentacular-like cells (S) between nests of neoplastic cells.

**FIGURE 8:**
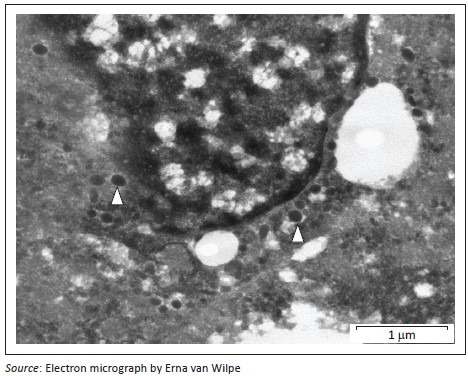
Transmission electron micrograph; renal paraganglioma. Intra-cytoplasmic (peri-nuclear) spherical, electron-dense-core neurosecretory granules within a neoplastic cell (white arrowheads).

A provisional diagnosis of bilateral neuroendocrine neoplasia was made with the main differential diagnosis being renal carcinoma. In order to obtain a definitive diagnosis, additional immunohistochemical stains were used including pan-cytokeratin, clone AE1/AE3 (broad-spectrum epithelial marker), chromogranin A (CgA) (marker for neuroendocrine differentiation), vimentin, clone 3B4 (labels cells of mesenchymal origin and melanocytes in normal and neoplastic tissues) and S100 (marker of glial cells in the central and peripheral nervous system, subpopulations of neurons, melanocytes, chondrocytes and adipocytes). Immunohistochemistry was performed according to standard staining protocols using an EnVision polymer-based immunodetection system (code no. K5007, DakoCytomation, Denmark) in the Pathology Section, Department of Paraclinical Sciences, Faculty of Veterinary Science, University of Pretoria. The neoplastic cells failed to label convincingly with the polyclonal rabbit anti-pan-cytokeratin antibody (code no. M3515, DakoCytomation) (Figure 3) and displayed multifocal widespread typical (cytoplasmic granular) positive labelling with the polyclonal rabbit anti-CgA antibody (code no. A0430, DakoCytomation) (Figure 4). The monoclonal mouse anti-vimentin antibody (code no. M7020, DakoCytomation) labelled the capsule, septa and thin stroma in the tumours, but there was no positive staining of the neoplastic cells (Figure 5). Rare sustentacular-like spindle-shaped cells located peripherally in neoplastic cell nests labelled characteristically (positive nuclear and cytoplasmic signal) with the polyclonal rabbit anti-S100 antibody (code no. Z0311, DakoCytomation) (Figure 6). Transmission electron microscopy revealed nests of neoplastic cells surrounded by collagen fibrils with embedded blood vessels and intermittent basal lamina, occasional sustentacular-like cells located between nests of tumour cells (Figure 7) and variable granular tumour cells with predominantly spherical electron-dense core neurosecretory-type granules (unfortunately only a few of which were obviously membrane-bound because of processing artefacts; Figure 8).

The immunohistochemical stains together with the ultrastructural findings were consistent with a diagnosis of extra-adrenal paragangliomas and not renal carcinomas. The essential diagnostic features of paraganglioma in this case included the CgA-positive tumour cells with ultrastructurally demonstrable cytoplasmic dense-core neurosecretory granules, and the observation of occasional S100-positive peripherally located sustentacular cells, which were also demonstrated ultrastructurally. In addition, the neoplastic cells were negative for the pan-cytokeratin (epithelial) marker, and also for vimentin, which detects the vast majority of mesenchymal tumours, as well as melanomas.

**FIGURE 9:**
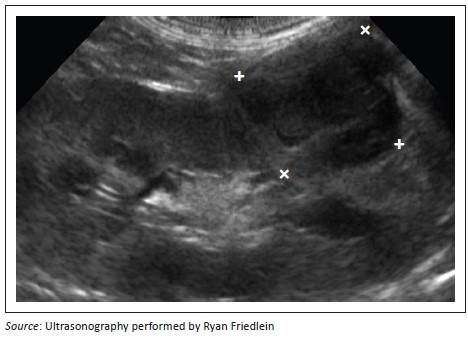
Left kidney sagittal view demonstrating a single heterogenous, hypoechoic nodule in the caudoventral aspect that measures 1.8 cm × 1.7 cm (between markers).

**FIGURE 10:**
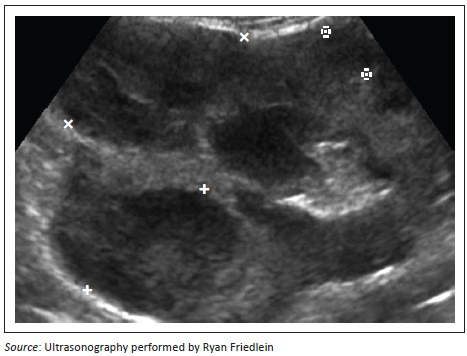
Right kidney transverse view demonstrating three irregularly rounded heterogenous hypoechoic nodules measuring 1.5 cm, 1.25 cm and 1 cm in diameter respectively (between markers).

Postoperatively the cat was sent home on prednisolone (Lenisolone, Aspen Pharmacare) at 1 mg/kg PO oid, potassium gluconate (Kaligel, Kyron) PO bid and omeprazole at 1 mg/kg PO oid. At the 2-week postoperative evaluation, the owner reported all vomiting had stopped and the cat was eating well. The blood pressure, urea, creatinine and electrolytes remained normal. Additional oral prednisolone was dispensed at 0.5 mg/kg PO oid and oral supplementation with potassium gluconate was continued. Repeated clinical examinations, biochemistry tests, blood pressure measurements and abdominal ultrasound examinations were performed every 4 weeks postoperatively. In addition to the above tests, echocardiography was performed to exclude a primary heart base neoplasm when a neuroendocrine tumour was suspected. Sixteen weeks postoperatively, full biochemistry, blood pressure measurements and thoracic radiographs remained normal; however, on subsequent abdominal ultrasonography evaluation, the nodules within the left kidney had increased in size to approximately 1.8 cm × 1.7 cm (Figure 9) and in number, and multiple similar nodules were also evident in the right kidney (Figure 10). The case was followed with serial evaluations of electrolyte and creatinine concentrations, blood pressure measurements as well as abdominal ultrasound evaluations. The cat continued on oral prednisolone at 0.5 mg/kg PO oid up until the writing of this report 24 weeks postoperatively. No biochemical or blood pressure alterations were evident during the stated postoperative period. Throughout the case follow-up, ultrasound evaluations demonstrated adrenal glands that remained within normal size limits. The adrenals were serially evaluated because of the concern for primary adrenal involvement, regional invasion and metastasis to the glands.

## Discussion

Veterinary literature regarding primary renal neoplasms in the cat is scarce, and information is generally limited to dated case reports and surveys of feline neoplasms (Engle & Brodey 1969; Henry et al. 1999; Whitehead 1967). The most common primary renal tumour is lymphosarcoma, which in most cases is bilateral, followed by renal carcinomas that are usually unilateral (Gabor, Malik & Canfield 1998; Henry et al. 1999). Additional primary renal tumours that have been demonstrated in cats include transitional cell carcinoma (Hanzlicek et al. 2012), cystadenoma (Mosenco et al. 2008), squamous cell carcinoma (Gomez Selgas, Scase & Foale 2014), adenoma (Clark & Wilson 1988), leiomyosarcoma (Evans & Fowlkes 2016); adenocarcinoma (Britt, Ryan & Howard 1985) and nephroblastoma (Henry et al. 1999), with none reported to be paragangliomas. Bilateral renal carcinomas were demonstrated in a case report in one cat (Steinberg & Thomson 1994).

Pheochromocytomas and extra-adrenal paragangliomas are tumours of neural crest-derived endocrine cells or organs known as paraganglia that exist throughout the autonomic nervous system (Tischler 2008). The World Health Organisation (WHO) classification system for human tumours reserves the term ‘pheochromocytoma’ for intra-adrenal tumours, whereas similar tumours in other locations are termed extra-adrenal paragangliomas, arising from sympathoadrenal and parasympathetic paraganglia (Tischler 2008). Extra-adrenal tumour locations are termed ‘paragangliomas’, irrespective of their sympathetic or parasympathetic origins (Mete et al. 2014). Pheochromocytomas and sympathetic paragangliomas frequently have associated clinical signs including tachyarrhythmias and hypertension (Maher & McNiel 1997), whereas the majority of parasympathetic paragangliomas are non-functional and non-symptomatic (Mete et al. 2014). The case above did not display sympathetic clinical signs (hypertension or tachyarrhythmias) and therefore may be of parasympathetic origin.

Extra-adrenal paragangliomas have been reported to arise from the pulmonary arch, left atrium, aortic body, extra- or paravertebral tissue, thoracic wall and along the sympathetic chain in dogs (Buchanan et al. 1998; Davis et al. 1997; Rizzo et al. 2008). In cats, they have been reported in the cauda equina, aortic arch and the abdominal cavity (Buchanan et al. 1998; Davis et al. 1997; Patnaik et al. 1990; Rizzo et al. 2008).

Because of their highly variable cytology and histopathology, pheochromocytomas and paragangliomas must be distinguished from a variety of endocrine and non-endocrine tumours (Tischler 2008). Differential diagnoses vary according to anatomic location, and distinctions may be made using immunohistochemical staining, with the most specific and reliable neuroendocrine marker being CgA (Aung et al. 2013; Tischler 2008). CgA readily distinguishes pheochromocytomas and paragangliomas from tumours that are not neuroendocrine (Tischler 2008). The staining is usually extensive, and caution is warranted in cases where tumours show little staining or do not stain at all (Tischler 2008).

In human pheochromocytomas and extra-adrenal paragangliomas, malignancy rates are generally low (ranging between 1% and 10%) and they metastasise late (Tischler 2008). The human WHO classification scheme defines malignancy of paragangliomas by the presence of metastases, specifically where paraganglionic tissue is not normally present (such as the liver and bone), and not by regional area invasion (McNicol 2011; Mete et al. 2014; Tischler 2008). The absence of detectable metastases, however, does not exclude their potential for future development (Tischler 2008). The case described here did not demonstrate local or distant metastasis during initial and subsequent evaluations followed over a 24-week postoperative period.

Primary neuroendocrine tumours of the kidney are rare and are frequently incorrectly diagnosed as other renal tumours such as renal cell carcinoma, mesonephric tumours or undifferentiated carcinoma (Aung et al. 2013). Renal paragangliomas are thought to arise from ectopic adrenal tissue or adrenal remnants located within the kidney (Bahar et al. 2014). In the present case report, the renal paragangliomas could have arisen from ectopic adrenal tissue within each kidney or as a result of metastasis from one kidney to the other. However, metastatic lesions would have also been expected elsewhere within the abdomen so the latter explanation is less likely. Renal paragangliomas can be difficult to distinguish clinically and histologically from renal cell carcinoma (Bahar et al. 2014), as was evident in this case following initial routine histopathology before the employment of additional immunohistochemical stains and TEM. Immunohistochemistry, although very useful in differentiating tumours of neuroendocrine origin, may not be helpful to diagnose malignant undifferentiated neoplasms; therefore, additional diagnostic modalities such as TEM are warranted (Rizzo et al. 2008).

In human medicine, primary renal paragangliomas are generally treated with a combination of renal bed irradiation and/or a multi-agent chemotherapy protocol (cyclophosphamide, vincristine and dacarbazine), but results are dismal using chemotherapy, with a complete response in tumour volume in 4% of patients and partial response in 37% of patients (Niemeijer et al. 2014; Yamamoto, Maeda & Mizoguchi 2007). There is insufficient literature regarding treatment options in the veterinary literature besides surgical removal, which is the only recommended treatment for all primary renal neoplasia besides lymphoma (Woldemeskel 2013). The above case was treated with prednisolone and a potassium supplement only, and a positive clinical response was demonstrated. The initial biochemistry tests that demonstrated hypochloraemia and hypokalaemia may be attributed to the clinical signs of anorexia and vomiting, or potassium loss via the kidneys. It is not clear why in the present case the cat showed the described clinical signs or if these signs were related to the renal neoplasia at all. It is certainly possible that another underlying clinical disease such as inflammatory bowel disease resulted in the anorexia and vomiting, hence the positive clinical response to prednisolone.

Despite clinical response to treatment with resolution of clinical signs, treatment with prednisolone appeared to have no effect on the actual paragangliomas. In contrast, the tumour appeared to progress locally within both kidneys, as witnessed by increased number and size of tumour lesions within both kidneys on ultrasound examination. Despite apparent progression of the tumour, no related clinical signs were seen and no evidence of deterioration of renal function was found throughout the study period. Blood pressures and serum urea and creatinine concentrations remained within normal limits throughout the entire period. An explanation for this would be enough residual and functional renal tissue was present to prevent clinical signs or the development of an azotaemia. Haematology, biochemistry, blood pressure and abdominal ultrasound examinations should be continued monthly to monitor the progression of the renal masses as well as the clinical signs of the cat.

Even though the clinical relevance of the paragangliomas in this cat is still uncertain and that treatment recommendations would not have differed if a renal cell carcinoma was diagnosed, this case report focuses on the difficulty of obtaining a conclusive diagnosis without the use of additional testing. It is evident from this case that additional testing, including IHC and TEM, is essential in order to achieve a definitive diagnosis of renal paragangliomas.

Limitations in the present case report include lack of catecholamine testing via the urine or serum (for evaluation of a catecholamine-secreting sympathetic phaeochromocytoma/paraganglioma) and the lack of urine-potassium fractional excretion testing (investigating the initial hypokalaemia).
